# Adiponectin Can Be an Early Predictable Marker for Type 2 Diabetes Mellitus and Nephropathy

**DOI:** 10.7759/cureus.27308

**Published:** 2022-07-26

**Authors:** Veluri Ganesh, Murugan M, Siva Prasad Palem

**Affiliations:** 1 Biochemistry, Aarupadai Veedu Medical College & Hospital, Vinayaka Mission’s Research Foundation (Deemed to be University), Salem, IND; 2 Biochemistry, JJM (Jagadguru Jayadeva Murugarajendra) Medical College and Hospital, Davanagere, IND

**Keywords:** nephropathy, microalbumin, egfr, type 2 diabetes mellitus, adiponectin

## Abstract

Background

Several studies have examined serum adiponectin concentrations in prediabetes, newly diagnosed type 2 diabetes mellitus (T2DM), and other types of diabetes associated with the risk of T2DM and diabetic nephropathy (DN); however, the results to date are inconclusive. An aim of the current study is to determine whether adiponectin is a useful marker for the earlier development of T2DM and DN.

Methodology

This cross-sectional study included 400 subjects. Among the subjects, 100 were prediabetes subjects, 200 were T2DM patients, and the remaining 100 were healthy controls. The biochemical and clinical parameters of all patients were analyzed and the data were recorded.

Results

The mean levels of adiponectin were significantly lower in prediabetic subjects than in healthy controls (3.22 ± 0.98, 5.36 ± 2.24, p = 0.0001**). Furthermore, the levels of adiponectin were significantly higher in both the groups of T2DM patients when compared to healthy controls (19.85 ± 3.31, 11.83 ± 3.01, and 5.36 ± 2.24, p = 0.0001**). In both diabetic groups, adiponectin was positively correlated with body mass index, glycated hemoglobin, insulin, homeostasis model assessment of insulin resistance, and microalbuminuria, while negatively correlated with estimated glomerular filtration rate. Interestingly, adiponectin had a reversed correlation in the prediabetic group.

Conclusion

Based on the results, the present study suggests that significantly decreased levels of serum adiponectin in prediabetic subjects might be used as a variable marker for T2DM. Moreover, adiponectin may useful for detecting the early onset of nephropathy, compared to microalbumin, as its concentration was significantly elevated in patients who were newly diagnosed with T2DM without nephropathy.

## Introduction

Hyperglycemia is a major risk factor for type 2 diabetes mellitus (T2DM), as it causes the improper secretion and activation of insulin. The incidence of it has tripled in the past few decades, especially in Asian countries. In fact, in 2019, there were 469 million people living with T2DM, a number which is predicted to cross 700 million by 2045 [1]. According to the American Diabetes Association (ADA), impaired fasting glucose and impaired glucose tolerance are indicative of the prediabetes stage, with 70% of prediabetes patients expected to progress to T2DM [2,3]. Factors such as a modern lifestyle, physical inactivity, and improper dietary habits are considered major risk factors for obesity, insulin resistance, and its associated metabolic diseases like T2DM [4]. Moreover, people with prediabetes are at a high risk of developing other complications, such as kidney, nervous, and cardiovascular diseases (CVD) [5]. Thus, it is imperative to diagnose prediabetes at an early stage and implement effective interventions before T2DM and its complications arise. 

Furthermore, diabetic kidney disease (DKD) is a prominent microvascular complication of T2DM and is the leading cause of end-stage renal disease (ESRD) and CVD [6]. Glomerular transient hyperfiltration, fibrosis, albuminuria, and diminished estimated glomerular filtration rate (eGFR) are common clinical manifestations of DN [7]. Microalbumin is the current early biomarker for nephropathy (normoalbuminuria: <29 mg/dL, microalbuminuria: 30-299 mg/dL, and macroalbuminuria: >300 mg/dL). It is also found in other conditions such as exercise, fever, congestive heart failure, hemodynamic stress, and urinary tract infections [8]. Given this context, its association with kidney failure remains unclear. Albuminuria is presumed to be determined by the severity of glomerular abnormalities, although this association is not strict in preceding the onset of proteinuria, as evidenced by iterated renal biopsies in T2DM patients [9]. Consequently, there is a necessity to evaluate noninvasive and more idiosyncratic biomarkers to predict the early onset of nephropathy in patients with T2DM. 

Adiponectin is the most abundant protein produced from white adipose tissue. It is expressed in tissues such as the liver, kidney, pancreas, skeletal muscle, bone, and glands [10]. It has been shown to ameliorate IR, oxidative stress, and inflammatory status using anti-diabetic, anti-oxidative, and anti-inflammatory properties for obesity-related diseases, lipid homeostasis, T2DM, and CVD [11]. The low levels of adiponectin associated with obesity-related IR, metabolic syndrome, and elevated levels of adiponectin in circulation inhibit gluconeogenic enzyme expression in T2DM mice [12]. Adenosine monophosphates activate protein kinase (AMPK), which, in turn, gets invigorated by adiponectin, resulting in the increased utilization of blood sugars and oxidation of fatty acids [13]. Additionally, adiponectin also plays a role in renal physiology by intercepting the excretion of proteins in urine. It does this by stimulating the AMPK and dwindling the predominant nicotinamide adenine dinucleotide phosphate oxidase 4 (Nox4) [14]. Various case-control, cohort, and prospective studies reported that decreased adiponectin levels associated with obesity, hypertension, dyslipidemia, blood sugar levels, and insulin resistance are potential risk factors for T2DM and its complications [15-17]. A systemic review and meta-analysis study reported elevated levels of adiponectin and a lower risk of T2DM [18], while another recent meta-analysis conclusively showed that hypoadiponectinemia was associated with the burgeoning of T2DM [19]. Additionally, another recent study reported that adiponectin receptors might be a component of insulin granules and therapies need to activate adiponectin receptors to reduce the risk of T2DM and its complications [20]. However, the results on the association between adiponectin and the incidence of T2DM remain unclear and conflicting, despite being many.

To bridge this gap, the present study aimed to determine an association between serum adiponectin concentrations in prediabetes and newly diagnosed T2DM and the risk of T2DM as well as nephropathy.

## Materials and methods

Study subjects 

**Figure 1:**
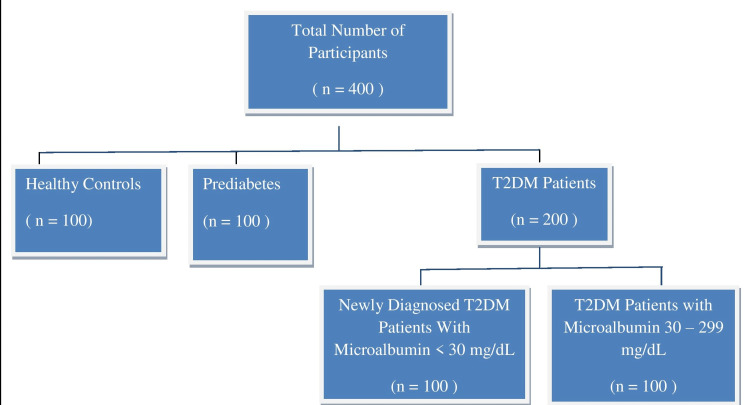
Selection of study participants T2DM, type 2 diabetes mellitus.

The present cross-sectional study had 400 participants. Among these, 300 participants were attending the outpatient clinic of General Medicine at Basaveshwara Medical College and Hospital, Chitradurga, Karnataka, India. These 300 participants were divided into two groups: prediabetes (n = 100) and T2DM (n = 200). The patients in these two groups were diagnosed as per the revised ADA criteria [21]. The rest of the 100 participants were matched by age, gender, and body mass index (BMI) and were considered healthy controls. As per the inclusion criterion, all the participants had to be aged between 30 and 70 years. They were all recruited on the basis of a follow-up survey that was conducted from April 2018 to December 2021. Subjects with prediabetes or impaired fasting glucose 100-125 mg/dL, glycated hemoglobin (HbA1c) 5.7-6.4%, and subjects with T2DM having fasting glucose >126 mg/dL and HbA1c >6.5% were included. These subjects were further categorized into four subgroups (Figure 1) and the adiponectin levels of the participants in all four subgroups were analyzed. Subjects who were smokers and alcoholics; subjects who were pregnant and lactating; subjects who had hypertension and other types of diabetes (type 1 diabetes mellitus, gestational DM); and subjects with a known history of infectious diseases, thyroid disorders, malignancy, cerebrovascular diseases, myocardial infarction, other types of kidney diseases, and mental illnesses were excluded. This study was approved by the institutional ethics committee, and it was conducted in accordance with the principles of Helsinki. Before enrolment, informed written consent was obtained from all the subjects. 

Specimen collection and laboratory evaluation

All the participants were instructed to observe a fast for 8-12 hours. Following this, 10 mL of fasting and 3 mL of postprandial venous blood specimens were withdrawn from their cubital fossa. The blood samples were centrifuged at 3000 RPM, and plasma and serum aliquots are stored at -80°C until a biochemical analysis was done. The plasma samples for the fasting and postprandial blood sugar were determined using the glucose oxidase-peroxidase method, while serum urea and creatinine concentrations were measured using glutamate dehydrogenase and the modified Jaffe’s method. We determined HbA1c and microalbumin by using a latex immunoturbidometric method, eGFR was calculated using the modification of diet in renal disease formula, and homeostasis model assessment of insulin resistance (HOMA-IR) was also calculated using insulin (µIU/mL) (glucose (mmol/L)/22.5. The Beckman Coulter auto analyzer was used to analyze all biochemical parameters. An enzyme-linked immunosorbent assay (ELISA, EUROIMMUN Auto Analyzer) was used to measure fasting insulin and serum adiponectin. 

Statistical analysis

The distribution of data was studied by using the Kolmogorov-Smirnov test and the data were expressed as mean ± standard deviation (SD). Repeated-measures analysis of variance (ANOVA) was used for comparison between all the study subjects. Pearson’s correlation analysis was used for the correlation between adiponectin concentration and other biochemical parameters. Univariate linear regression analysis was done to assess the relationship between eGFR, microalbumin, and serum adiponectin. Receiver operating characteristic (ROC) curves were constructed to study the diagnostic accuracy of the markers to identify diabetes and DN in prediabetes and T2DM with normoalbuminuria group when compared to the healthy controls. A “p” Value <0.05 was considered statistically significant. All statistical analyses were performed using IBM Statistical Package for the Social Studies (SPSS) windows version 20.0 (SPSS Inc, Chicago, IL, USA) and Medcalc (Version 12.1, Ostend, Belgium) and Microsoft Excel spreadsheets. 

## Results

Demographic, biochemical, and clinical characteristics of prediabetes and healthy individuals are listed in Table 1. The mean levels of BMI, fasting blood sugar (FBS), post-prandial blood sugar (PPBS), creatinine, HbA1c, eGFR, insulin, HOMA-IR, and microalbumin were statistically strongly significant (p = 0.0001**) and eGFR concentrations shown were moderately significant in prediabetes when compared to controls (p = 0.001*). There were no statistical differences in age (45.51 ± 6.87) and urea (23.24 ± 9.92) in participants with prediabetes when compared to controls (44.74 ± 8.12, 20.58 ± 2.47), respectively (p values were 0.0470 and 0.028). The serum adiponectin concentrations were significantly decreased in the prediabetes (3.22 ± 0.98) group when compared to the healthy individuals (5.36 ± 2.24, p = 0.0001**).

**Table 1:** Comparison of means of the anthropometric, biochemical, microalbumin, and serum adiponectin data among prediabetes and healthy controls. **Highly significant, *Significant, †NS - not significant at the 0.05 probability level. n, number; p, probability; M:F, male and female ratio; BMI, body mass index; FBS, fasting blood sugar; PPBS, post-prandial blood sugar; HbA1C, glycosylated hemoglobin; HOMA-IR, homeostasis model assessment of insulin resistance; eGFR, estimated glomerular filtration rate.

Parameter	Healthy Controls (n = 100)			Prediabetes (n = 100)			p-Value
Age (years)	44.74	±	8.12	45.51	±	6.87	0.470^†^
Gender (M:F)	48	:	52	45	:	55	
BMI (kg/m^2^)	20.49	±	2.47	31.29	±	3.28	0.0001**
FBS (mg/dL)	86.28	±	7.49	113.40	±	6.83	0.0001**
PPBS (mg/dL)	113.60	±	17.38	137.31	±	10.18	0.0001**
Serum urea (mg/dL)	20.58	±	6.74	23.24	±	9.92	0.028^†^
Serum creatinine (mg/dL)	0.94	±	0.20	1.21	±	0.21	0.0001**
HbA1C (%)	4.31	±	0.77	5.91	±	0.43	0.0001**
eGFR (mL/min)	109.62	±	27.22	98.61	±	17.22	0.001*
Insulin (µIU/mL)	6.27	±	1.69	12.23	±	2.00	0.0001**
HOMA-IR	2.14	±	0.48	3.44	±	2.60	0.0001**
Microalbumin (mg/dL)	5.18	±	2.08	12.02	±	3.39	0.0001**
Serum adiponectin (μg/mL)	5.36	±	2.24	3.22	±	0.98	0.0001**

Table 2 illustrates the anthropometric and clinical characteristics of the newly diagnosed T2DM patients with normoalbuminuria as well as T2DM patients with microalbuminuria. It was found that T2DM patients with microalbuminuria had a significantly higher age and lower BMI than the newly diagnosed T2DM patients with normoalbuminuria (p = 0.0001**). Moreover, the table shows that the concentrations of FBS, PPBS, urea, creatinine, HbA1c, HOMA-IR, microalbumin, and serum adiponectin are statistically elevated and highly significant in T2DM patients with microalbuminuria when compared to newly diagnosed T2DM patients with normoalbuminuria (p < 0.0001**). There were significantly decreased levels of eGFR and insulin in the T2DM patients with the microalbuminuria group, compared to newly diagnosed T2DM patients with normoalbuminuria (p < 0.0001**). 

**Table 2:** Comparison of means of the descriptive and clinical characteristics among two groups of T2DM patients. **Highly significant at the 0.05 probability level. n, number; p, probability; M:F, male and female ratio; BMI, body mass index; FBS, fasting blood sugar; PPBS, post-prandial blood sugar; HbA1C, glycosylated hemoglobin; HOMA-IR, homeostasis model assessment of insulin resistance; eGFR, estimated glomerular filtration rate; T2DM, type 2 diabetes mellitus.

Parameter	Newly Diagnosed T2DM With Normoalbuminuria (n = 100)			T2DM With Microalbuminuria (n = 100)			p-Value
Age (years)	47.17	±	5.65	54.12	±	7.43	0.0001**
Gender (M:F)	83	:	17	47	:	53	-
BMI (kg/m^2^)	24.35	±	3.55	22.85	±	4.29	0.0001**
FBS (mg/dL)	156.39	±	32.80	178.07	±	19.34	0.0001**
PPBS (mg/dL)	169.82	±	24.38	271.42	±	52.83	0.0001**
Serum urea (mg/dL)	32.88	±	8.73	80.58	±	20.66	0.0001**
Serum creatinine (mg/dL)	1.32	±	0.29	7.75	±	1.23	0.0001 **
HbA1C (%)	7.06	±	0.91	9.18	±	1.17	0.0001**
eGFR (mL/min)	78.64	±	22.06	59.13	±	11.58	0.0001**
Insulin (µIU/mL)	18.31	±	5.86	13.15	±	4.54	0.0001**
HOMA-IR	4.84	±	1.67	8.94	±	3.04	0.0001**
Microalbumin (mg/dL)	13.66	±	7.30	178.54	±	40.56	0.0001**
Serum adiponectin (μg/mL)	11.83	±	3.01	19.85	±	3.31	0.0001**

Association between adiponectin and clinical parameters in prediabetes

To further investigate the relationship between adiponectin and clinical parameters, both were tabulated (Table 3). The results showed an inverse association between adiponectin and BMI, HbA1c, insulin, and microalbumin (r = -0.397, -0.457, -0.439, and -0.433, respectively; p = 0.0001**). Moreover, the adiponectin concentrations were found to be inversely associated and had no statistical significance with HOMA-IR (r = -0.099; p = 0.163). Additionally, adiponectin had a significant direct association with eGFR (r = 0.138; p = 0.05*). 

**Table 3:** Correlation of serum adiponectin with other clinical parameters of nephropathy. **Highly significant, *significant, †NS - not significant. r, correlation coefficient; T2DM, type 2 diabetes mellitus; BMI, body mass index; HbA1c, glycosylated hemoglobin; HOMA-IR, homeostasis model assessment of insulin resistance; eGFR, estimated glomerular filtration rate.

Parameter	Prediabetes		Newly Diagnosed T2DM With Normoalbuminuria		T2DM With Microalbuminuria	
	r	p	r	p	r	p
BMI	-0.397	0.0001**	0.437	0.0001**	0.205	0.004*
HbA1c	-0.457	0.0001**	0.655	0.0001**	0.829	0.0001**
Insulin	-0.439	0.0001**	0.570	0.0001**	0.564	0.0001**
HOMA-IR	-0.099	0.163†	0.532	0.0001**	0.793	0.0001**
eGFR	0.138	0.05*	-0.347	0.0001**	-0.688	0.0001**
Microalbumin	-0.433	0.0001**	0.573	0.0001**	0.871	0.0001**

Correlation between adiponectin and other indexes among T2DM subgroups divided by microalbumin 

Adiponectin levels in newly diagnosed T2DM patients with normoalbuminuria were found to be strongly correlated with BMI, HbA1c, insulin, HOMA-IR, and microalbumin (r = 0.437, 0.655, 0.570, 0.532, and 0.573, respectively; p = 0.0001**) and it was found to be significantly negatively correlated to eGFR (r = -0.347; p = 0.0001**). In T2DM patients with microalbuminuria, the adiponectin concentrations were positively correlated with HbA1c, insulin, HOMA-IR, and microalbumin (r = 0.829, 0.564, 0.793, and 0.871; p = 0.0001**). Additionally, we observed a moderate correlation between adiponectin and BMI (r = 0.205; p = 0.004*). Moreover, serum adiponectin in T2DM patients with microalbuminuria was inversely correlated with eGFR (r = -0.688; p = 0.0001**) (Table 3).

The diagnostic utility of the BMI, HbA1c, insulin, HOMA-IR, and serum adiponectin for the early detection of T2DM was compared in prediabetes and healthy controls by ROC analysis as shown in Table 4. As described, among these, BMI, HbA1c, insulin, and adiponectin showed a statistically significant area under the curve (AUC) with a sensitivity range from 93, 94, 88, and 88 and specificity from 100, 92, 98, and 68, respectively (p-value is 0.0001**). Additionally, we observed that HOMA-IR showed no significant AUC with a low sensitivity of 33 and a high specificity of 100 (p = 0.0990).

**Table 4:** ROC analysis of prediabetes and healthy controls **Highly significant, †NS - not significant. ROC, receiver operating characteristic; BMI, body mass index; HbA1c, glycosylated hemoglobin; HOMA-IR, homeostasis model assessment of insulin resistance; AUC, area under the curve; CI, confidence interval.

Parameter	AUC	95% CI for AUC	Sensitivity (%) 95% CI	Specificity (%) 95% CI	p-Value
BMI	0.985	0.957-0.997	93	100	0.0001**
HbA1c	0.983	0.954-0.996	94	92	0.0001**
Insulin	0.984	0.955-0.996	88	98	0.0001**
HOMA-IR	0.569	0.497-0.638	33	100	0.0990†
Serum adiponectin	0.791	0.728-0.845	88	68	0.0001**

## Discussion

**Figure 2:**
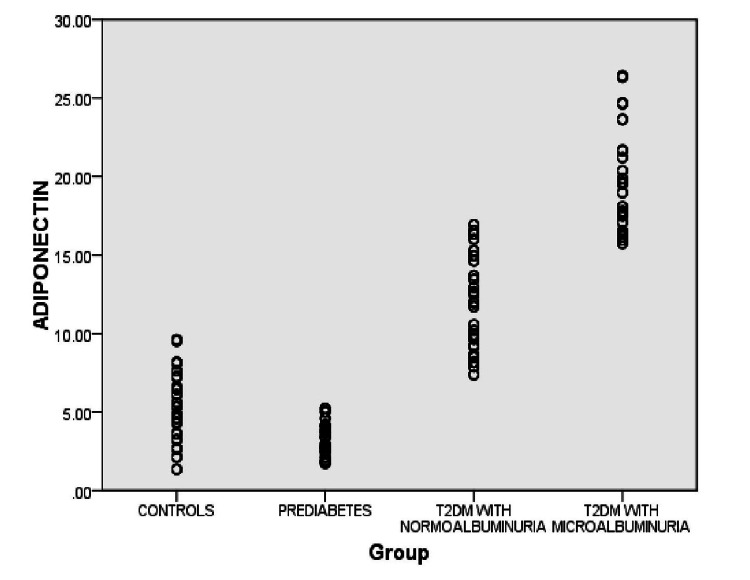
Comparison of serum adiponectin concentrations in all the study participants. T2DM, type 2 diabetes mellitus.

The adiponectin concentrations have shown a significant decrease among prediabetic patients when compared to healthy controls. We also noted significantly elevated adiponectin concentrations, particularly in patients with newly diagnosed T2DM. Our study was the first study to evaluate serum adiponectin levels and their association with prediabetes, T2DM, and DN among the Indian population (Figure 2). 

Gadadharan V et al. reported that obesity has become a major factor to escalate the accumulation of visceral body fat in the adipose tissue. The factors behind this were found to be advanced lifestyle changes (physical inactivity, food habits), western food habits (low intake of fruits and vegetables and consumption of a high-fat diet and beverages consisting of high sugars), stress, smoking, alcoholism, and metabolic and genetic factors, which lead to systemic and metabolic diseases [22]. Jiang Y et al. suggested that adiponectin levels are a significant marker of incident prediabetes and the development of T2DM [23]. On the other hand, Anindita B et al. reported reduced adiponectin concentrations as an independent risk factor for the progression to prediabetes and T2DM, which might be helpful for developing experimental models as well as identifying biomarkers [4]. In our study, we found a significant inverse association between serum adiponectin and BMI in prediabetic subjects (p = 0.0001**). This states that hypoadiponectinemia may pose the risk of T2DM. 

**Figure 3:**
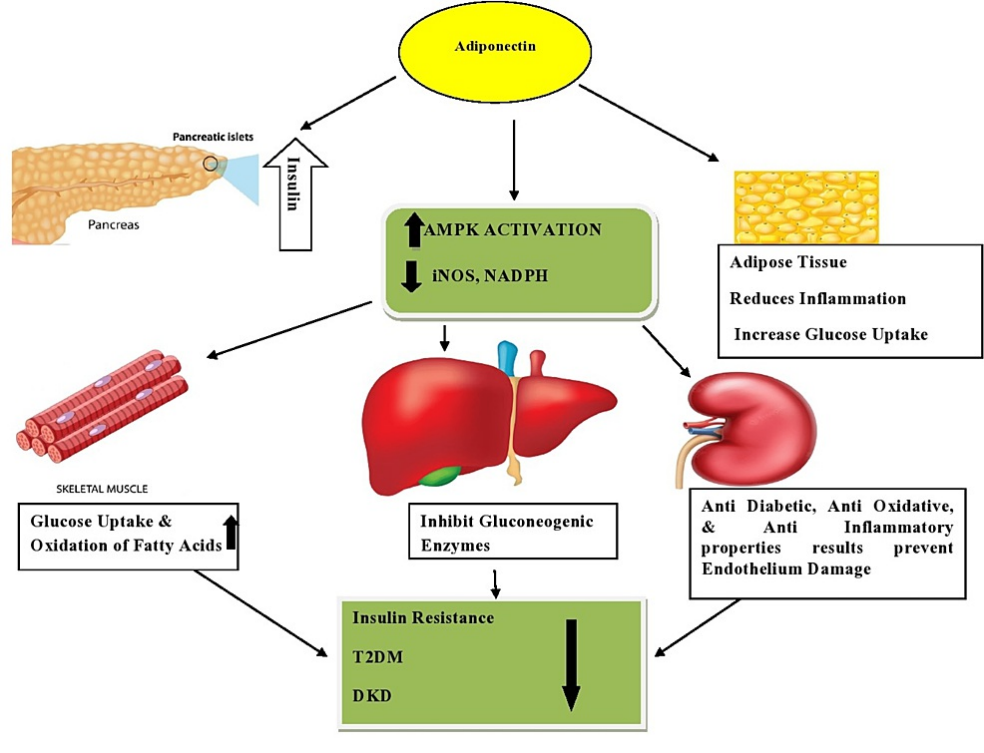
Role of adiponectin in T2DM and DKD AMPK, AMP-activated protein kinase; iNOS, inducible nitric oxide synthase; NADPH, nicotinamide adenine dinucleotide phosphate; T2DM, type 2 diabetes mellitus; DKD, diabetic kidney disease.

**Figure 4:**
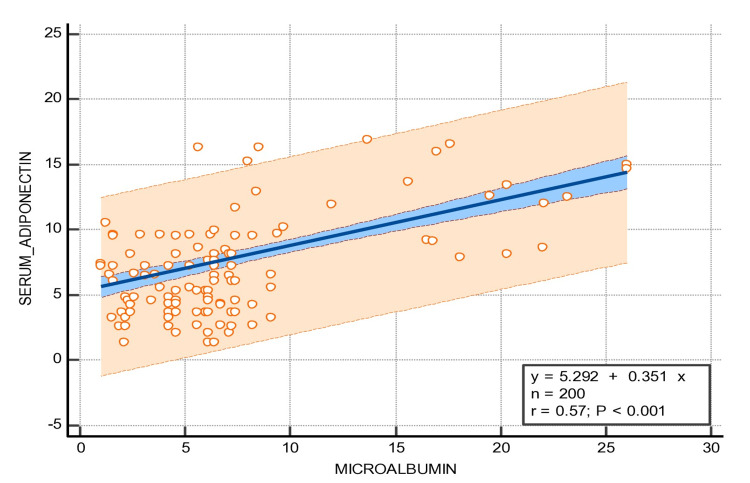
Regression analysis of serum adiponectin with microalbumin.

**Figure 5:**
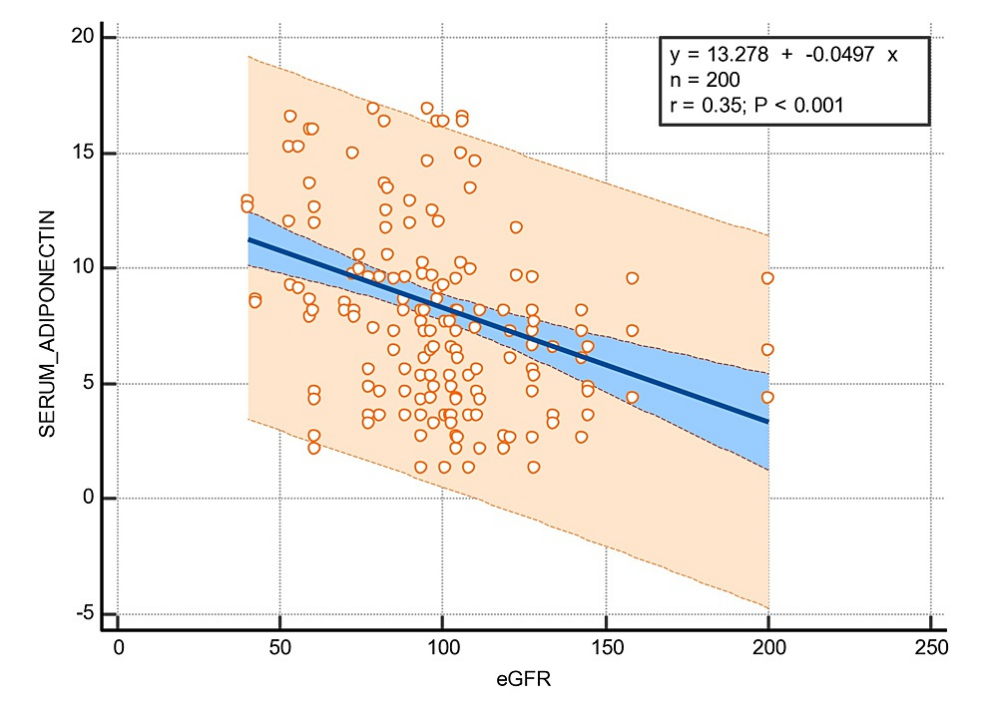
Regression analysis of serum adiponectin with eGFR. eGFR, estimated glomerular filtration ratio.

Liu W et al. reported that adiponectin plays an essential role in improving insulin sensitization, which prevents the development of T2DM as well as the damage of certain types of tissues through its anti-diabetic, anti-oxidative, and anti-inflammatory properties [24]. Dongqing Z et al. reported that statistically elevated levels of plasma adiponectin might be useful as a (novel) marker for diabetes, as well as DN [25] (Figure 3). Kuo IC et al. reported significantly higher adiponectin levels. These levels were directly associated with the urinary albumin creatinine ratio while having an inverse association with eGFR. Moreover, the monitoring of adiponectin levels was independently associated with a greater likelihood of developing early DN [26]. Similarly, in our study, the mean of the serum adiponectin levels was found to be statistically elevated among newly diagnosed T2DM patients with normoalbuminuria as well as among T2DM patients with microalbuminuria. Moreover, we focused on the correlation of serum adiponectin with the clinical parameters of nephropathy and it was found to be a significant positive correlation with microalbumin (Figure 4) and negative correlation with eGFR (p ≤ 0.001*) (Figure 5). 

Temesgen F et al. posited that T2DM patients were more prone to developing nephropathy due to persistent albuminuria and progression to ESRD. Diagnosis of the early stages of nephropathy is essential as early interventions can slow the loss of function and decrease adverse outcomes for kidney [27]. At present, microalbumin is used as the earliest marker for DN. However, it has been reported that it is a conventional and not a sensitive and specific biomarker for DN, since a large proportion of kidney dysfunction and several similar confounding issues are elevated in non-diabetic subjects during exercise and in conditions such as urinary tract infection and illness as well [28,29]. Recent studies have demonstrated that serum adiponectin levels are significantly elevated in T2DM patients with normoalbuminuria when compared to healthy individuals. Hence, adiponectin may be a suitable marker for an earlier, specific, and accurate prediction of nephropathy. 

Additionally, in this study, we estimated the diagnostic utility of the BMI, HbA1c, insulin, HOMA-IR, eGFR, microalbumin, and serum adiponectin for the early detection of DN by comparing newly diagnosed T2DM patients with normoalbuminuria and healthy controls using ROC curve analysis, as shown in Table 5. Among these measures, the BMI, HbA1c, insulin, HOMA-IR, eGFR, microalbumin, and adiponectin showed a statistically significant AUC with a sensitivity range value of 74, 100, 91, 94, 60, 71, and 97 and a specificity value of 80, 100, 100, 100, 87, 92, and 84, respectively (p = 0.0001**).

**Table 5:** ROC curve analysis of newly diagnosed T2DM patients with normoalbuminuria and healthy controls. **Highly significant. BMI, body mass index; HbA1c, glycosylated hemoglobin; HOMA-IR, homeostasis model assessment of insulin resistance; eGFR, estimated glomerular filtration rate; AUC, area under the curve; CI, confidence interval; ROC, receiver operating characteristic.

Parameter	AUC	95% CI for AUC	Sensitivity (%) 95% CI	Specificity (%) 95% CI	p-Value
BMI	0.824	0.764-0.874	74	80	0.0001**
HbA1c	1.000	0.982-1.000	100	100	0.0001**
Insulin	0.936	0.893-0.966	91	100	0.0001**
HOMA-IR	0.983	0.953-0.996	94	100	0.0001**
eGFR	0.804	0.742-0.856	60	87	0.0001**
Microalbumin	0.861	0.805-0.906	71	92	0.0001**
Serum adiponectin	0.968	0.933-0.988	97	84	0.0001**

Limitations

Since this is a single-center study, further multiple-center and follow-up studies are required to strengthen the importance of adiponectin for the early prediction of T2DM and DN. 

## Conclusions

The present study suggests that significantly decreased levels of serum adiponectin in prediabetic subjects can be a potential marker for T2DM. Moreover, adiponectin levels may serve as a better predictive marker for the early onset of nephropathy than microalbumin levels, since adiponectin concentrations were found to be significantly elevated in patients who were newly diagnosed with T2DM without nephropathy and also inversely associated with eGFR levels.

## References

[1] Huang K, Liang Y, Ma Y, Wu J, Luo H, Yi B (2022). The variation and correlation of serum adiponectin, nesfatin-1, IL-6, and TNF-α levels in prediabetes. Front Endocrinol (Lausanne).

[2] Tabák AG, Herder C, Rathmann W, Brunner EJ, Kivimäki M (2012). Prediabetes: a high-risk state for diabetes development. Lancet.

[3] Lai H, Lin N, Xing Z, Weng H, Zhang H (2015). Association between the level of circulating adiponectin and prediabetes: a meta-analysis. J Diabetes Investig.

[4] Banerjee A, Khemka VK, Roy D, Poddar J, Roy TK, Karnam SA (2017). Role of serum adiponectin and vitamin D in prediabetes and diabetes mellitus. Can J Diabetes.

[5] Milman S, Crandall JP (2011). Mechanisms of vascular complications in prediabetes. Med Clin North Am.

[6] Kravets I, Mallipattu SK (2020). The role of podocytes and podocyte-associated biomarkers in diagnosis and treatment of diabetic kidney disease. J Endocr Soc.

[7] Kowalski A, Krikorian A, Lerma EV (2014). Diabetic nephropathy for the primary care provider: new understandings on early detection and treatment. Ochsner J.

[8] Jim B, Ghanta M, Qipo A (2012). Dysregulated nephrin in diabetic nephropathy of type 2 diabetes: a cross sectional study. PLoS One.

[9] Fioretto P, Mauer M (2007). Histopathology of diabetic nephropathy. Semin Nephrol.

[10] Sun Y, Xun K, Wang C, Zhao H, Bi H, Chen X, Wang Y (2009). Adiponectin, an unlocking adipocytokine. Cardiovasc Ther.

[11] Gong X, You L, Li F (2021). The association of adiponectin with risk of pre-diabetes and diabetes in different subgroups: cluster analysis of a general population in south China. Endocr Connect.

[12] Kadowaki T, Yamauchi T, Kubota N, Hara K, Ueki K, Tobe K (2006). Adiponectin and adiponectin receptors in insulin resistance, diabetes, and the metabolic syndrome. J Clin Invest.

[13] Yamauchi T, Kamon J, Minokoshi Y (2002). Adiponectin stimulates glucose utilization and fatty-acid oxidation by activating AMP-activated protein kinase. Nat Med.

[14] Sharma K, Ramachandrarao S, Qiu G (2008). Adiponectin regulates albuminuria and podocyte function in mice. J Clin Invest.

[15] Selthofer-Relatić K, Kibel A, Delić-Brkljačić D, Bošnjak I (2019). Cardiac obesity and cardiac cachexia: is there a pathophysiological link?. J Obes.

[16] Duncan BB, Schmidt MI, Pankow JS (2004). Adiponectin and the development of type 2 diabetes: the atherosclerosis risk in communities study. Diabetes.

[17] Abdella NA, Mojiminiyi OA (2018). Clinical applications of adiponectin measurements in type 2 diabetes mellitus: screening, diagnosis, and marker of diabetes control. Dis Markers.

[18] Li S, Shin HJ, Ding EL, van Dam RM (2009). Adiponectin levels and risk of type 2 diabetes: a systematic review and meta-analysis. JAMA.

[19] Liu C, Feng X, Li Q, Wang Y, Li Q, Hua M (2016). Adiponectin, TNF-α and inflammatory cytokines and risk of type 2 diabetes: a systematic review and meta-analysis. Cytokine.

[20] Fisman EZ, Tenenbaum A (2014). Adiponectin: a manifold therapeutic target for metabolic syndrome, diabetes, and coronary disease?. Cardiovasc Diabetol.

[21] American Diabetes Association (2020). 10. Cardiovascular disease and risk management: standards of medical care in diabetes-2020. Diabetes Care.

[22] Vijayakumar G, Manghat S, Vijayakumar R (2019). Incidence of type 2 diabetes mellitus and prediabetes in Kerala, India: results from a 10-year prospective cohort. BMC Public Health.

[23] Jiang Y, Owei I, Wan J, Ebenibo S, Dagogo-Jack S (2016). Adiponectin levels predict prediabetes risk: the Pathobiology of Prediabetes in A Biracial Cohort (POP-ABC) study. BMJ Open Diabetes Res Care.

[24] Liu W, Zhou X, Li Y (2020). Serum leptin, resistin, and adiponectin levels in obese and non-obese patients with newly diagnosed type 2 diabetes mellitus: a population-based study. Medicine (Baltimore).

[25] Zha D, Wu X, Gao P (2017). Adiponectin and its receptors in diabetic kidney disease: molecular mechanisms and clinical potential. Endocrinology.

[26] Kuo IC, Wu PH, Lin HY (2019). The association of adiponectin with metabolic syndrome and clinical outcome in patients with non-diabetic chronic kidney disease. PLoS One.

[27] Fiseha T (2015). Urinary biomarkers for early diabetic nephropathy in type 2 diabetic patients. Biomark Res.

[28] Rigalleau V, Lasseur C, Raffaitin C (2007). Normoalbuminuric renal-insufficient diabetic patients: a lower-risk group. Diabetes Care.

[29] Jain S, Rajput A, Kumar Y, Uppuluri N, Arvind AS, Tatu U (2005). Proteomic analysis of urinary protein markers for accurate prediction of diabetic kidney disorder. J Assoc Physicians India.

